# Rapid identification of *Pseudomonas aeruginosa* by pulsed-field gel electrophoresis

**DOI:** 10.1080/13102818.2014.981065

**Published:** 2014-11-18

**Authors:** Samy Selim, Iman El Kholy, Nashwa Hagagy, Sahar El Alfay, Mohamed Abdel Aziz

**Affiliations:** ^a^ Department of Clinical Laboratory Sciences, College of Applied Medical Science, Aljouf University, Sakaka, Saudi Arabia; ^b^ Department of Botany, Faculty of Science, Suez Canal University, Ismailia, Egypt; ^c^ Clinical Pathology Department, Ain Shams University Specialized Hospital, Ain Shams University, Cairo, Egypt

**Keywords:** *Pseudomonas aeruginosa*, pulsed-field gel electrophoresis, Egypt

## Abstract

Twenty clinical *Pseudomonas aeruginosa* isolates recovered from patients admitted to The General Hospital in Ismailia Governorate (Egypt) were examined in this study. We analysed *P. aeruginosa* ATCC 9027 (as a control strain) and 19 of the isolates after digestion with *SpeI* restriction endonuclease. After this we conducted a pulsed-field gel electrophoresis (PFGE) and typed the obtained 10 unique patterns, designated as A, A1, B, B1, C, C1, D, D1, E and F. We evaluated the genetic relatedness between all strains, based on ≥87% band identity. As a result, the isolates were grouped in the 10 clusters as follows: patterns A, A1, B, B1, C contained two strains each and patterns C1, D, D1, E contained a single strain each; the five remaining strains were closely related (genomic pattern F). One isolate belonged to antibiotype ‘b’. The genotype patterns of the *P. aeruginosa* ATCC 9027 control strain and isolate no. 11 were closely related and had two different antibiotypes ‘d’ and ‘c’, respectively.

## Introduction

*Pseudomonas aeruginosa* is a Gram-negative rod which does not ferment glucose. This micro-organism can be rarely observed as a part of the human microflora in healthy individuals. *P. aeruginosa* is the most important human pathogen from its genus. It may cause infections, particularly, in patients with cystic fibrosis, bronchiectasis, neutropenia, acquired immune deficiency syndrome (AIDS), burns and in those with metabolic, haematologic or malignant diseases.[1] *P. aeruginosa* is a major reason for nosocomial infections in patients who are in intensive care units, causing mainly ventilator-associated pneumonia, surgical site infections (SSIs), urinary tract infections and sepsis. It is also a poor prognosis marker, as it is associated with a higher mortality rate.[2]

During the last decade, traditional phenotypic typing methods for epidemiologic and outbreak studies have been replaced by molecular methods.[3] Pulsed-field gel electrophoresis (PFGE) typing is one of the most useful discriminating methods to type *Pseudomonas* spp.[3,4] Although DNA-based techniques have proved successful in epidemiological studies of *P. aeruginosa*,[5] they are time consuming and expensive and require specific equipment.[6] That is why clinical laboratories commonly use antibiotic susceptibility and biochemical tests in routine analyses.[7–9] Quantitative analysis of antibiograms by comparing disk zone sizes is considered useful for nosocomial infection control in some cases.[10] Distinct biotypes can be determined by biochemical tests in combination with production of pigment and haemolysis. Although *P. aeruginosa* gives a uniform response in some biochemical tests used for identification purposes, the variability of the response to other tests can be used to distinguish clinical isolates based on different profiles (biotypes). In agar cultures, most *P. aeruginosa* strains form typical blue–green colonies due to the characteristic pigments pyocyanin and fluorescein. Some *P. aeruginosa* strains, however, can form colonies with a distinct colour due to other pigments, or can form even non-pigmented colonies.[7,11]

In the present study, 20 clinical isolates of *P. aeruginosa* from hospitalized patients were evaluated for epidemiological relatedness. Two phenotypic methods (antibiotic susceptibility and biochemical patterns) and PFGE genotyping of DNA were used. Based on the obtained phenotypic and genotypic data, the accuracy of antibiotic profiles was evaluated and possible clonal relationships among the clinical isolates were analysed.

## Materials and methods

### Microbiological methods

Seven hundred patients from Public Ismailia Hospital (Egypt) were investigated. Samples from urine, wound discharge and burns were collected from these patients. Samples were handled by sterile swabs in clean dry and sterile containers. All collected samples were transferred into laboratory conditions within a few hours. The samples were streaked onto Cetrimide agar (Scharlau, Spain) and Nutrient agar and were incubated overnight under aerobic conditions at 37 °C to obtain individual colonies. The suspected *P. aeruginosa* colonies, which had green fluorescent colour, were picked up and purified. Gram-stained films of the single colonies were prepared and examined microscopically. Gram-positive micro-organisms (if present) were excluded from subsequent analyses. Gram-negative rods were further identified.

In this study, we analysed 20 clinical isolates of *P. aeruginosa* recovered from patients and one *P. aeruginosa* ATCC (9027) strain used as the control. *P. aeruginosa* was identified on the basis of several characteristics, such as Gram staining, colony morphology, odour, production of pigment, oxidase test, growth on Kligler's medium slants (K/K),[12] urease production, growth on Cetrimide agar [13] and confirmation by API 20 NE kit test.

The susceptibility tests were performed using the agar disk diffusion method, according to the guidelines of the National Committee for Clinical Laboratory Standards.[14] There are several different classes of antimicrobial agents commonly used and available for treatment of *P. aeruginosa.* In this study, we used aztreonam (ATM 30 μg), ceftazidime (CAZ 30 μg), ciprofloxacin (CIP 5 μg) and imipenem (IPM 10 μg). We classified the isolates as susceptible or resistant, based on the size of the zone of inhibition. In the susceptibility typing, the strains were classified as antibiotype ‘a’ (fully susceptible to antibiotics); antibiotype ‘b’ (multiresistant to three types of antibiotics); antibiotype ‘c’ (multiresistant to two types of antibiotics) and antibiotype ‘d’ (susceptible to three antibiotics, with a diameter of the clear zone ranging between 1.5 and 3.5 cm).

Biotyping was performed by observation of haemolysis after growth on blood agar medium (Oxoid) for 24 h under transmitted light. Haemolytic isolates were divided into two groups, according to the observation of α or *ß* haemolysis. Production of pigment was determined on Mueller–Hinton medium (Scharlau, Spain) after incubation for 24 h at 35 °C and the pigmented colonies were classified according to their colour.

### DNA genomic typing by PFGE

Macrorestriction analysis by PFGE of DNA was performed according to USA Centers for Disease Control and Prevention (CDC) highly standardized PFGE protocols [15] for Gram-negative rods with some minor modifications.[16] Bacterial suspensions were prepared from individual bacterial colonies directly obtained from cultures incubated overnight on Mueller–Hinton agar. The suspensions were adjusted to a concentration of 10^9^ CFU/mL (colony-forming units per millilitre), which is equal to 1:1.5 NTU (Nephelometric Turbidity Unit) in ethylenediaminetetraacetic acid (EDTA)–saline buffer (75 mmol/L NaCl and 25 mmol/L EDTA, pH 7.5). The cell suspension was mixed with an equal volume of 1% low-melting point seaKem Gold Agarose (Cambrex Bio Sciences Rockland, Inc.) and was allowed to solidify in a 100 μL plug mould. The agarose plug was incubated for 24 h at 37 °C in 500 μL of lysis buffer (6 mmol/L Tris–HCl (pH 7.6), 0.1 mol/L EDTA, 1 mol/L NaCl, 0.5% Brij®58 (polyoxyethylene (20) cetyl ether; Sigma), 0.4% sodium deoxycholate, 0.5% sodium lauryl sarcosine and 1 mg/mL lysozyme). Next, the lysis buffer was replaced with 500 μL of proteinase K buffer (1% sodium lauryl sarcosine, 0.5 mol/L EDTA (pH 9) and proteinase K (50 μg/mL; Sigma)) and this solution was incubated with gentle shaking at 50 °C for 20 h. The plugs were then washed four times for 30 min at 37 °C with 10 mL of Tris–EDTA buffer (10 mmol/L Tris–HCl (pH 8) and 1 mmol/L EDTA). One-third of a slice of each plug was cut and incubated for 18–20 h with 30 U of *SpeI* (Bio-Rad Laboratories) in the restriction buffer (Promega Buffer). DNA restriction fragments were separated by PFGE by using a CHEF DR III apparatus (Bio-Rad, Richmond, CA, USA) at 14 °C, 6 V/cm, for 20 h, with a time switch of 2–40 s. A *Salmonella* serotype Branderup strain (H9812) ladder (Bio-Rad Laboratories) restricted with *XbaI* was used as a universal size marker.[17] The gel was stained with ethidium bromide and visualized with the Gel-Doc system (Bio-Rad Laboratories). According to the criteria by Tenover et al. [3], isolates were considered to be genetically indistinguishable or identical if the restriction fragments had the same number of bands and the corresponding bands were with identical apparent size. Therefore, these isolates were designated as genomic pattern A. Isolates were considered to be closely related if their PFGE patterns showed differences in two or three of their bands, consistent with a single genetic event, and those isolates were classified as genomic pattern A1. Isolates were possibly related if their PFGE patterns showed differences in four to six of their bands, associated with two independent genetic events, and those isolates were classified as genomic pattern A2. Isolates were considered to be unrelated if their PFGE patterns showed differences in seven or more of their bands, corresponding to three or more independent genetic events, and were designated as type A, A1, B, B1, C, C1, D, D1, E and F.

## Results and discussion

Of the 20 clinical isolates, 10% were obtained from urine and the other 90% were obtained from different surgical wounds. Pigment production was observed in 16 of the isolates, most of which displayed the characteristic blue–green, yellowish-green and yellow colour (Table 1). All isolates were haemolytic and most of them demonstrated β-haemolysis (85.7%). The susceptibility typing classified only one isolate (No. 12) as antibiotype ‘a’; one isolate (No. 6) as antibiotype ‘b’; one isolate (No. 11) as antibiotype ‘c’ and the rest of the isolates (85.7%) as antibiotype ‘d’.

**Table 1.** Major characteristics, antibiotic susceptibility and biochemical profiles observed in *Pseudomonas aeruginosa* clinical isolates.

			Zone of inhibition (cm)					
Isolate no.	Source	Antibiotype	CAZ	IPM	ATM	CIP	Pigment production	Haemolysis
1	Wound	d	0	3	2.2	3	Yellow	β
2	Wound	d	0	3.2	2.2	2.8	Yellow	β
3	Wound	d	0	3	1.5	2.5	Yellow	β
4	Wound	d	0	3.9	1.7	2.8	Blue–green	β
5	Urine	d	0	2.5	3	2	None	α
6	Wound	b	0	2.3	0	0	Yellow	β
7	Wound	d	0	2.7	2.8	3.2	Blue–green	β
8	Wound	d	0	2.8	1.8	2.7	Yellow	β
9	Urine	d	0	3.6	2.4	2.7	Rosy-brown	β
10	Wound	d	0	2.8	2.6	3.2	Yellow	β
11	Wound	c	0	4.6	0	4.2	None	β
12	Wound	a	1.7	3	2	3	None	β
13	Urine	d	0	2.7	2.8	3.2	No growth	α
14	Wound	d	0	3	1.8	2.5	Blue–green	β
15	Wound	d	0	1.7	2	2.5	No growth	β
16	ATCC 9027	d	0	2.7	2.1	2.5	Yellowish-green	**–**
17	Wound	d	0	3.4	2	3.6	Pale-yellow	β
18	Wound	d	0	3.4	2.4	2.9	Yellow	β
19	Wound	d	0	3.4	2.5	3	Yellowish-green	β
20	Wound	d	0	3	2.4	2.4	Yellowish-green	β
21	Wound	d	0	3.3	2.8	2.9	Yellowish-green	β

Note: CAZ – ceftazidime (30 μg), IPM – imipenem (10 μg), ATM – aztreonam (30 μg), CIP – ciprofloxacin (30 μg); β – complete blood haemolysis, α – partial blood haemolysis. Antibiotype ‘a’ (fully susceptible to antibiotics), ‘b’ (multiresistant to three types of antibiotics), ‘c’ (multiresistant to two types of antibiotics) and ‘d’ (susceptible to three types of antibiotics with clear zone diameter ranging between 1.5 and 3.5 cm).

**Figure 1.**
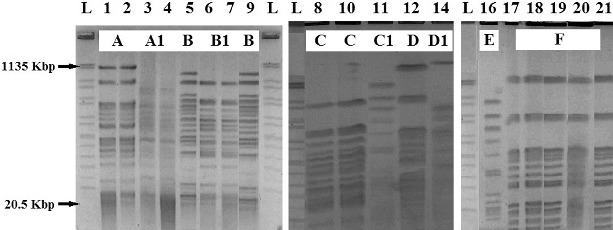
DNA genomic patterns of *Pseudomonas aeruginosa* digested with *SpeI*. PFGE patterns A, A1, B, B1, C, C1, D, D1, E and F are shown. Note: L: DNA ladder; Lanes 1–21: isolates (see Table 1); Lane 16 – *P. aeruginosa* ATCC 9027 control strain.

*SpeI* digestion of the 19 *P. aeruginosa* isolates from different patients and *P. aeruginosa* ATCC 9027 control strain were typed using PFGE (one isolate was eliminated during the analysis), giving 10 unique patterns (designated as pattern types A, A1, B, B1, C, C1, D, D1, E and F; Figure 1). When we compared the genetic relatedness (defined as ≥87% band identity) of the strains, they were grouped in the following 10 clusters: two strains in pattern A; two in pattern A1; two in pattern B, two in pattern B1, two in pattern C; patterns C1, D, D1, E contained single isolate each and the five remaining strains were closely related (genomic pattern F). We took the PFGE profiles as standard. We observed that genotype B1 included two distinct antibiotypes, ‘b’ and ‘d’. Genotypes A, A1, B and C contained two isolates and two different antibiotypes each. Patterns D1 and E contained one isolate. Genotype F included five isolates, which belonged to antibiotype ‘d’. Genotypes C1 and D contained a single isolate each and they had their own unique antibiotypes ‘c’ and ‘a’, respectively. The genotype pattern of the *P. aeruginosa* ATCC 9027 control strain and isolate no. 11 were closely related and had two different antibiotypes ‘d’ and ‘c’, respectively.

Our results from DNA restriction enzyme analysis by PFGE (molecular typing) demonstrated that most *P. aeruginosa* isolates belonged to distinct genotypes, in agreement with previous reports [7] that this method has a higher discriminatory power than the phenotypic methods (antibiotyping and biotyping). Our results are also in support of the observation made by Freitas and Barth [7] that, although isolates with unique susceptibility profiles displayed distinct genotypes, the difference in antibiotype may not guarantee clonal distinction. This is due to the fact that many isolates of the same genotype may display distinct susceptibility profiles, which could question the adequacy of antibiotyping for typing purposes.[17,18] As a whole, it is not surprising that the discriminatory power of susceptibility tests is low, since it depends on the number of types defined by a method and the relative frequencies of these types.[7,19] Phenotypic methods can be used for initial screening of isolates which may be further typed by a more discriminatory test.[7,20–22] However, biotype, antibiotype or a combination of both may in some cases be insufficient for screening and typing of different genotypes.[7,14] The isolation of the same bacteria from patients in the same unit may be of help to detect an outbreak.[22]

However, it has been shown that susceptibility profiles are not acceptable as a presumption of relatedness or distinction among *P. aeruginosa* isolates, indicating that a clonal relation can be identified only through DNA typing, such as PFGE-based typing.[7,14] This could explain why macrorestriction analysis of DNA by PFGE has become a widespread technique for typing bacterial isolates, which is further supported by the general applicability of this method to any species [23] and its powerful discriminatory potential for the classification of isolates.[22,23] For example, Römling and Tümmler [24] reported that the typeability, i.e., the percentage of strains that could be assigned a type, was 100%. PFGE is also a relatively inexpensive, fast and reproducible DNA typing tool for effective epidemiological surveillance of potentially transmissible *P. aeruginosa* isolates.[25] Hence, macrorestriction analysis has long been considered the method of choice for typing of *P. aeruginosa* isolates.[26,27]

## Conclusions

The results from this study demonstrate the power of PFGE as a suitable, relatively inexpensive, fast, reproducible and highly discriminatory DNA-typing tool for analysis of potentially transmissible *P. aeruginosa* isolates.
